# Should we enlarge the indication for kidney biopsy in patients with diabetes? The pro part

**DOI:** 10.1093/ckj/sfad266

**Published:** 2023-10-26

**Authors:** Loreto Gesualdo, Marco Fiorentino, Francesca Conserva, Paola Pontrelli

**Affiliations:** Renal, Dialysis and Transplantation Unit, Department of Precision and Regenerative Medicine and Ionian Area (DIMEPRE-J), University of Bari, Bari, Italy; Renal, Dialysis and Transplantation Unit, Department of Precision and Regenerative Medicine and Ionian Area (DIMEPRE-J), University of Bari, Bari, Italy; Renal, Dialysis and Transplantation Unit, Department of Precision and Regenerative Medicine and Ionian Area (DIMEPRE-J), University of Bari, Bari, Italy; Renal, Dialysis and Transplantation Unit, Department of Precision and Regenerative Medicine and Ionian Area (DIMEPRE-J), University of Bari, Bari, Italy

**Keywords:** chronic kidney disease, classification, diabetes, phenotypes, precision medicine

## Abstract

Diabetic nephropathy (DN) and non-diabetic renal diseases (NDRD) represent intricate challenges in diagnosis and treatment within the context of the global diabetes epidemic. As the prevalence of diabetes continues to escalate, effective management of renal complications becomes paramount. Recent advancements in comprehending the multifaceted nature of renal damage, fueled by insights from histopathological investigations, offer unprecedented prospects for refining diagnostic strategies and customizing therapeutic interventions. Renal biopsies have emerged as indispensable tools for unraveling the diverse phenotypes of renal damage in diabetes. The pioneering study by Mazzucco identified three classes of renal damage in type 2 diabetes patients: classical diabetic glomerulosclerosis (DN), vascular and ischemic glomerular changes (NDRD), and other glomerulonephritides in the presence (DN + NDRD, mixed forms) or absence of DN (NDRD). The prevalence of these classes varies widely in published studies, influenced by factors such as ethnicity, geography and selection criteria for renal biopsy. Moreover, the international Renal Pathology Society consensus classification system has stratified the classical diabetic nephropathy into progressive categories of renal impairment, a breakthrough that aids in prognostication. Histopathological scrutiny, particularly the intricate correlation between glomerular and tubulointerstitial lesions, contributes profoundly to enhancing our grasp of the phenotype's heterogeneity. This amplified comprehension holds the potential to steer personalized treatment strategies. Cutting-edge interventions, encompassing sodium-glucose cotransporter 2 inhibitors, mineralocorticoid receptor antagonists and anti-endothelin receptor agents, are broadening the arsenal against renal injury in diabetes. When combined with the profound insights garnered from histopathological, omics, imaging and clinical data, these therapeutic avenues promise a transformative shift towards precision-driven care paradigms. Collaborative efforts uniting researchers, clinicians and patients are indispensable for propelling our knowledge of diabetic renal damage and ameliorating patient outcomes. The fusion of histopathological, omics and imaging findings into clinical decision-making harbors the potential to customize interventions and optimize care for individuals grappling with diabetes-associated renal complications. Furthermore, groundbreaking initiatives like the iBeat Study within the BEAt-DKD (Biomarker Enterprise to Attack Diabetic Kidney Disease) project (https://www.beat-dkd.eu/), elucidating distinct phenotypes of renal damage within diabetes, underscore the imperative necessity of integrating histopathological data into the broader framework of diabetic renal management.

## RENAL DAMAGE COMPLEXITY IN DIABETES

Diabetes mellitus, a global health concern, has reached epidemic proportions, with an estimated 643 million people worldwide projected to be living with the condition by 2030 [1]. As diabetes prevails, its complications cast a long shadow, affecting multiple organ systems. Among these complications, chronic kidney disease (CKD), often referred to as diabetic kidney disease (DKD), remains a significant concern, impacting a substantial portion of diabetic individuals. Predictions suggest that more than 150 million diabetes patients may develop DKD by 2030 [2].

Among DKD, real diabetic nephropathy (DN), characterized by microangiopathic changes, poses a substantial threat, eventually progressing to end-stage kidney disease over an indeterminate time span [3]. The classical trajectory of DN, initially observed in type 1 diabetes patients and later extrapolated to type 2 diabetes (T2DM), encompasses an initial phase of microalbuminuria preceding the emergence of overt nephropathy, marked by macroalbuminuria and a gradual decline in glomerular filtration rate (GFR) [4]. However, it is evident that T2DM patients may exhibit varied clinical presentations with unpredictable and divergent disease courses, presenting challenges in accurate diagnosis and management.

DKD encompasses various renal damage forms among diabetic patients, which can be further categorized as: classical diabetic glomerulosclerosis (DN), vascular and ischemic glomerular changes (NDRD), and other glomerulonephritides in the presence (DN + NDRD, mixed forms) or absence of DN (NDRD) [2]. The prevalence of these classes varies widely in published studies, influenced by factors such as ethnicity, geography and the selection criteria for renal biopsy [3].

The variability in renal involvement patterns among T2DM patients, coupled with the potential occurrence of glomerulonephritides independent of diabetes, complicates the accurate diagnosis of DN, NDRD and the mixed forms [5]. This challenge has driven nephrologists to rely on renal biopsies as a prerequisite for precise diagnosis, while also prompting the search for non-invasive biomarkers that can aid in distinguishing among the different forms. Renal biopsy, however, has not achieved universal acceptance due to concerns surrounding potential complications and the prevailing notion among nephrologists that CKD in the context of diabetes is predominantly attributed to DN [6].

## EXPANDING THE ROLE OF RENAL BIOPSY IN DIABETIC RENAL DAMAGE DIAGNOSIS AND TREATMENT

Renal biopsy is still a cornerstone diagnostic tool in nephrology, allowing clinicians to unravel the complex and heterogeneous nature of renal diseases [5, 6]. In the context of diabetes, where renal complications often exhibit a diverse spectrum of phenotypes, the value of renal biopsy becomes paramount. The traditional paradigm of classifying renal damage based solely on clinical markers is no longer sufficient to address the intricacies of DN vs NDRD vs DN + NDRD. As we delve into the multifaceted landscape of renal damage, it becomes evident that renal biopsy plays a pivotal role in refining clinical diagnosis, guiding therapeutic decisions and ultimately improving patient outcomes.

## UNLOCKING THE PHENOTYPIC COMPLEXITY

The notion that all diabetic patients develop a uniform nephropathy is now being challenged [2, 3, 7]. The classification of DKD based solely on albuminuria and estimated GFR has proven inadequate to grasp the full breadth of renal damage. In this scenario, novel clinical phenotypes of DKD have been identified in recent years. The normoalbuminuric renal impairment encompasses a significant proportion of DKD patients, according to the RIACE (Renal Insufficiency And Cardiovascular Events) study [8]; this phenotype is characterized by the predominance of macrovascular and tubule-interstitial changes compared with the albuminuric DKD phenotype and, consequently, by a high cardiovascular risk. The limited histological information reported throughout the literature describes a high heterogeneity of lesions, supporting the importance of the histopathological evaluation of renal biopsy as the key to unlocking the phenotypic complexity within DKD. This approach allows for the identification of distinct subtypes, each with its unique molecular underpinnings and therapeutic implications. Recent research, such as the iBeat Study within the BEAt-DKD (Biomarker Enterprise to Attack Diabetic Kidney Disease) project (https://www.beat-dkd.eu/), has unraveled the existence of multiple phenotypes within DKD [9]. In the current phase of the project the aim is to identify novel non-invasive biomarkers specifically able to discriminate the different trajectories of the disease.

## BEYOND CLINICAL MARKERS: MOLECULAR INSIGHTS

While clinical markers provide valuable information, they often fail to encapsulate the intricate molecular mechanisms driving renal damage in diabetes [10–12]. Histopathological analysis not only confirms clinical observations but also provides essential insights into the molecular signatures underlying renal damage. For instance, the identification of specific immune complexes and inflammatory infiltrates in renal biopsies can guide the selection of immunomodulatory therapies in patients with immunological-vascular damage, such as immunoglobulin A nephropathy, membranous nephropathy and focal segmental glomerulosclerosis. This molecular understanding extends our therapeutic armamentarium, enabling more targeted and effective interventions and improving long-term outcomes.

## PRECISION MEDICINE: TAILORING TREATMENT TO HISTOPATHOLOGY

The era of precision medicine is upon us, and renal biopsy is pivotal in this approach. As we navigate the landscape of DN, NDRD and DN + NDRD, the need for tailored therapeutic strategies becomes evident. The clinical response to interventions can vary significantly among patients, and this variability can be attributed to the underlying histopathological profile [13] other than to the different comorbidities due to the diabetic milieu. Histopathology provides the roadmap for tailoring treatment and reducing the progression toward end-stage kidney disease. For example, patients with a predominant vascular component might benefit from therapies targeting endothelial dysfunction, while those with immune-mediated damage might require immunosuppressive agents. This individualized approach maximizes therapeutic efficacy while minimizing adverse effects.

## REDEFINING DISEASE CLASSIFICATION

The traditional classification of renal diseases has been reshaped by the insights gathered from histopathological analysis. The concept of NDRD, initially dismissed within the diabetic cohort, has gained prominence. Distinct from DN and NDRD, DN + NDRD encompasses a myriad of conditions, ranging from hypertensive nephropathy to glomerulonephritides. The misdiagnosis of NDRD as DN is not uncommon based on clinical evidences and can lead to suboptimal treatment outcomes. Renal biopsy serves as the pivot point for distinguishing between these entities, thereby guiding appropriate therapeutic strategies.

To define the molecular mechanisms that can affect the different regions in the kidney thus leading to different kinds of renal damage, spatial anchoring is essential to understand the relationship between cells and function, since renal cells interact with each other within a cosmos of unique microenvironments.

In very recent years, spatial transcriptomic technologies have enabled the localization of whole-transcriptome mRNA expression, correlation of mRNA to histology, measurement of *in situ* changes in expression across time and even subcellular localization of transcripts within a complex tissue such as the kidney.

Despite impressive advances, bulk RNA-seq can only measure gene expression in cell mixtures, without revealing the transcriptional heterogeneity and spatial patterns of single cells. These innovations continue to aid in the development of human cellular atlases of the kidney, the reclassification of diseases, the understanding of their progression trajectory and the identification of important therapeutic targets [14].

## INTEGRATING NEW THERAPIES: UNLEASHING THE POTENTIAL

The evolving understanding of the diverse phenotypes within diabetic renal damage has profound implications for therapeutic approaches. Alongside traditional renin–angiotensin–aldosterone system (RAAS) inhibition, new therapeutic avenues are emerging. Sodium-glucose cotransporter 2 inhibitors, mineralocorticoid receptor antagonists and anti-endothelin receptors hold promise in further refining treatment strategies. These therapies, when applied selectively based on histopathological phenotypes, have the potential to enhance the outcomes beyond conventional RAAS blockade. The identification of metabolically driven, vascular and immune-mediated phenotypes through renal biopsy can guide the selection of these novel therapies, maximizing their efficacy and limiting adverse effects.

## THE PATH FORWARD: INTEGRATING BIOMARKERS AND OMICS TECHNOLOGIES

While histopathological analysis remains indispensable, ongoing research in the BEAt-DKD project is focused on integrating emerging imaging biomarkers and omics technologies into the diagnostic landscape. The identification of specific biomarkers that correlate with distinct histopathological subtypes and imaging features could enhance diagnostic accuracy and minimize the need for invasive procedures. Omics technologies offer a holistic view of molecular alterations, enabling the identification of multiple pathogenic mechanisms leading to the different phenotypes and novel therapeutic targets. These advancements, when synergized with histopathology, promise to revolutionize our approach to diabetic renal damage.

## THE NEED FOR CONSENSUS AND VALIDATION

As we advocate for the wider adoption of renal biopsy in diabetic renal damage diagnosis, it is crucial to acknowledge the challenges that lie ahead. Establishing a standardized histopathological, omics and imaging classification system that aligns with clinical outcomes and therapeutic responses is imperative. The collaborative efforts of clinicians, researchers and pathologists are essential in devising a unified framework. Additionally, validation studies are crucial to demonstrate the clinical utility and prognostic value of this new classifications. These endeavors will solidify the role of renal biopsy in shaping treatment paradigms for diabetic renal damage.

## CONCLUSION: PIONEERING PERSONALIZED CARE

In conclusion, the argument advocating for an expanded indication of kidney biopsy in patients with diabetes is grounded in the imperative need for precise diagnosis in light of the continuously evolving landscape of disease manifestations and treatment paradigms. The heterogeneity characterizing renal complications in T2DM necessitates a shift toward a more individualized approach to patient care. Kidney biopsy emerges as an invaluable tool to navigate the intricate maze of DKDs, guiding therapeutic decisions and laying the foundation for innovative non-invasive diagnostic methods. Embracing this broader perspective allows us to provide more effective care tailored to the complex needs of diabetic patients, ultimately enhancing their overall well-being. In the pursuit of elevated patient care standards, renal biopsy not only serves as an irreplaceable tool in unraveling the multifaceted nature of diabetic renal damage but also acts as a mirror reflecting the systemic impact of microvascular complications. For instance, patients with microvascular damage are at risk of developing diabetic retinopathy, while those with ischemic vascular lesions face a heightened risk of myocardial infarction. Therefore, achieving a precise histological classification is pivotal in comprehending and managing patients with DKDs.

The transition from a one-size-fits-all medical approach to personalized medicine is underpinned by the wealth of information unlocked through renal biopsy. As we stride toward an era of healthcare tailored to individual needs, the role of renal biopsy becomes increasingly indispensable, shedding light not only on the renal intricacies but also on the broader spectrum of systemic repercussions in diabetes and beyond.
